# Effects of Twelve Weeks of Square Stepping Exercises on Physical and Cognitive Function and Plasma Content of SMP30: A Randomised Control Trial

**DOI:** 10.3390/geriatrics10010022

**Published:** 2025-02-07

**Authors:** Juan Manuel Franco-García, Jorge Pérez-Gómez, Antonio Castillo-Paredes, Pedro Cosme Redondo, Jorge Rojo-Ramos, Noelia Mayordomo-Pinilla, Santos Villafaina, Mari Carmen Gómez-Álvaro, Maria Melo-Alonso, Jorge Carlos-Vivas

**Affiliations:** 1 Health, Economy, Motricity and Education (HEME) Research Group, Faculty of Sport Sciences, University of Extremadura, 10003 Cáceres, Spain; jmfrancog@unex.es (J.M.F.-G.); jorgepg100@unex.es (J.P.-G.); 2 Grupo AFySE, Investigación en Actividad Física y Salud Escolar, Escuela de Pedagogía en Educación Física, Facultad de Educación, Universidad de Las Américas, Santiago 8370040, Chile; 3 Cell Physiology Research Group, Department of Physiology, University of Extremadura, 10003 Cáceres, Spain; pcr@unex.es; 4 Promoting a Healthy Society Research Group (PHeSO), Faculty of Sport Sciences, University of Extremadura, 10003 Cáceres, Spain; jorgerr@unex.es; 5 BioErgon Research Group, Faculty of Sports Sciences, University of Extremadura, 10003 Cáceres, Spain; nmayordo@alumnos.unex.es; 6 Grupo de Investigación en Actividad Física, Calidad de Vida y Salud (AFYCAV), Departamento de Didáctica de la Expresión Musical, Plástica y Corporal, Facultad de Ciencias del Deporte, Universidad de Extremadura, Av. de la Universidad s/n, 10003 Cáceres, Spain; svillafaina@unex.es (S.V.); maricarmengomezal@unex.es (M.C.G.-Á.); mmeloa@unex.es (M.M.-A.); 7 Physical Activity for Education, Performance and Health (PAEPH) Research Group, Faculty of Sport Sciences, University of Extremadura, 10003 Cáceres, Spain; jorgecv@unex.es

**Keywords:** ageing, electroencephalography, executive cognitive function, physical fitness, quality of life

## Abstract

Background: Ageing and sedentary lifestyles affect physical and cognitive function and markers of frailty, increasing the risk of falls in older adults and affecting their quality of life. The aim of this study was to evaluate the effects of a Square Step Exercise programme on physical and cognitive function and plasma SMP30 levels for the prevention of falls in older adults. Methods: A randomised controlled trial was designed with 44 participants assigned to an experimental group (SSE group) and a control group. The SSE group performed SSE sessions twice a week for three months, with a follow-up in the fourth month. The assessments of physical function included tests such as the Four-Square Step Test, Brisk Walking and its dual-task variant, Time Up and Go and its imagined and dual-task variants, 30 s Sit-to-Stand and its dual-task and imagined variants and a 6 min walking test. Questionnaires were also used to assess the risk and fear of falling. Resting EEG activity was also recorded to assess electrocortical brain activity. SMP30 levels were measured by Western blotting. Results: The SSE group showed significant improvements compared to the control group in the Four-Square Step Test (*p* < 0.001), Brisk Walking (*p* < 0.05) and reduction in the fear of falling (*p* < 0.001) after the training programme, but these adaptations were not maintained one month after the programme ended (*p* < 0.05). No significant changes were observed in the remaining variables of physical function, cognitive function, fall risk questionnaire, EEG activity or plasma levels of SMP30 compared to the control group (*p* > 0.05). Conclusions: The SSE programme showed efficacy in improving balance, gait speed and reducing fear of falling in older adults but did not show improvement over the control group in other areas of physical or cognitive function or plasma SMP30 levels for fall prevention.

## 1. Introduction

The world’s population is ageing rapidly, with the number of people aged over 80 expected to triple by 2050 [1]. Ageing is associated with the progressive degeneration of organ systems leading to reduced bone and muscle mass, reduced mobility and increased dependency due to frailty [2,3]. Lifestyle factors such as poor diet, physical inactivity and harmful habits play a significant role in accelerating these age-related problems [4,5].

One of the most pressing issues associated with ageing is the increased risk of falls [6], which has a significant impact on the daily lives of older adults and places a significant burden on healthcare systems [7]. Approximately one in three individuals over the age of 65 fall each year, with 20–30% of these falls requiring hospitalisation [8,9,10]. By 2030, the incidence of falls in older people is predicted to increase by 30% with estimated healthcare costs of more than USD 50 billion [11,12]. Falls can have serious consequences such as injury, reduced mobility, loss of autonomy, social isolation and depression, all of which contribute to a decline in quality of life [13,14]. Addressing this problem requires a multifaceted approach that promotes healthy ageing and targets fall prevention.

To achieve effective fall prevention, it is important to address the causes of falls and their associated risk factors [15]. One of the key factors is the age-related decline in sensorimotor systems, resulting in muscle weakness, decreased muscle strength, reduced joint mobility, sarcopenia and balance problems which increase the risk of falls [16,17,18]. Physical function in older people can reverse and reduce these risk factors and is one of the most important health markers associated with physical activity [19]. Physical activity has a direct effect on the physical fitness of older people, improving the performance of everyday tasks by improving balance, gait speed, muscle strength and cardiorespiratory fitness [20,21,22,23]. Strength training, using weights or elastic bands, increases muscle strength and the ability to perform the activities of daily living by preventing or improving sarcopenia [24,25]. Aerobic and multi-component programmes have also been shown to improve gait and reduce the risk of falls [23]. These interventions contribute to healthy ageing and the prevention of dependency, although further research is needed, as well as the development of more versatile and comprehensive programmes that are safe and easy to deliver in community groups.

Emerging research also suggests that certain biomarkers may serve as predictors of fall risk, providing valuable insights into the physiological changes associated with ageing [26]. For example, changes in brain-derived neurotrophic factor and its role in neuroplasticity may provide insights into the cognitive and sensorimotor impairments associated with falls [27]. Similarly, elevated levels of inflammatory markers such as C-reactive protein and interleukin-6 have been associated with reduced physical function and increased frailty [28,29]. Biomarkers reflecting muscle degradation, such as creatine kinase and myostatin levels, may also be indicators of sarcopenia and reduced muscle strength [30,31]. In addition, reduced vitamin D levels have been linked to impaired muscle function and reduced bone density, both of which contribute to the risk of falls [32]. In this context, Senescence Marker Protein 30 (SMP30) is becoming increasingly important as it may play a key role in healthy ageing and the prevention of chronic diseases [33].

This protein was discovered in 1978 and takes its name from its binding to Ca^2+^ [34]. In humans, its coding gene has been identified on chromosome Xp 11.3-Xp 11.23 [35]. Studies show that it is involved in two main areas: the prognosis of liver cancer, as a marker [36], and ageing. The association of this protein with ageing is justified by physiological regulation, as reduced expression may be associated with the loss of bone mass, dysfunction of lipid metabolism and impaired glucose tolerance, as well as involvement in vitamin C synthesis [37]. Understanding the behaviour of SMP30 in ageing may be relevant to the development of exercise interventions that are effective in the prevention of disease and its associated health problems. In this context, the effects of exercise programmes on SMP30 levels have been reported. One study showed that 12 weeks of whole-body vibration training in postmenopausal women resulted in a significant 27.7% increase in plasma SMP30 levels [38]. However, another study in frail older adults following a multi-component exercise programme showed no significant change in plasma SMP30 levels [39]. These findings highlight the importance of future studies to further our understanding of the effects of different exercise programmes on SMP30 levels.

In addition to the physical and biological functions of age in the risk of falling, it is important to consider the effects of brain function. As the brain ages, it undergoes changes in structure and neural activity [40,41,42,43]. These changes can lead to a decrease in brain activity, with an increase in slow wave frequencies and a decrease in higher frequencies, which can affect cognitive and sensorimotor function [43,44]. As many activities of daily living, such as walking, require a smooth interaction between physical and cognitive abilities [45,46,47,48,49], ageing can affect the ability to perform these activities safely, increasing the risk of falls in older people [50]. Therefore, it is essential to implement a comprehensive training approach that addresses both physical and cognitive aspects to effectively prevent falls [51,52]. While existing programmes tend to focus predominantly on one of these aspects, the approach developed in this study explicitly integrates physical–cognitive interaction and seeks to overcome these limitations [21].

Research into physical–cognitive interactions has used electroencephalography (EEG) as a tool to record electrical signals from brain cells [53]. It has become a user-friendly tool, making it more accessible for use in research related to physical activity and exercise [54]. The frequency of these waves varies depending on the characteristics of the physical activity, making the study of these changes relevant to the modification of motor-related cognitive impairment in ageing [55]. Some studies have shown that activity patterns in the beta band are associated with the anticipation of loss of balance and a corrective response [56]. In addition, it has been found that performing a secondary task while walking results in a longer stride, suggesting that extending the stride requires less attentional resources [57]. Similarly, research integrating cognitive tasks with physical exercise has shown changes in beta waves during dual tasking and has been associated with improved postural control, thus reducing the risk of falls [58]. However, EEG changes because of an exercise intervention are often elusive and difficult to replicate consistently, as the brain’s response to exercise can vary depending on factors such as intensity, programme duration and individual participant characteristics, which can make it difficult to obtain consistent results [59]. Nevertheless, the use of EEG remains relevant and promising as a tool to further explore the neurophysiological mechanisms underlying the physical–cognitive interaction, providing valuable information for optimising fall prevention programmes.

In this context, the effectiveness of Square Step Exercise (SSE) training has been highlighted as a potentially promising intervention. These exercises focus on strengthening muscles, improving coordination and increasing stability in everyday activities [60]. Shigematsu and Okura introduced SSE as a simple training strategy that involves performing steps in multidirectional patterns of varying complexity [61]. In addition, several studies have supported the beneficial effects of SSE in reducing the risk of falls in older adults [61,62], highlighting its relevance for health promotion and injury prevention in this population. Compared to other training methods for fall prevention, for which there is little scientific evidence, SSE has been shown to be as effective as balance training and Tai Chi Chuan [62,63]. Unlike these training methods, SSE adds an important cognitive component by requiring participants to memorise and reproduce step patterns. Also, unlike Tai Chi, which can require lengthy learning and correct technique, SSE is relatively simple to implement and can be easily adapted to different skill levels and available space. This facilitates its use in community or clinical settings with limited resources. However, more research is needed on the benefits of implementing SSE in fall prevention. Therefore, the aim of this study is to analyse and evaluate the effect of an SSE training programme on physical and cognitive function and plasma SMP30 levels for the prevention of falls in older adults.

## 2. Materials and Methods

### 2.1. Study Design

A randomised controlled trial with one-to-one allocation of experimental and control groups was conducted. The Consolidated Standards of Reporting Trials Statement (CONSORT) methodology was used [64].

### 2.2. Sample Size Calculations

For the present study, the required sample size was calculated using G*Power (version 3.1.9.4) [65]. An a priori power analysis was performed for a repeated-measures ANOVA, within–between interaction, including two groups and three measurement points. The parameters used were as follows: medium effect size (f = 0.25), significance level (α = 0.05), statistical power (1 − β = 0.95), correlation among repeated measures (0.7) based on the FSST outcomes [66] and nonsphericity correction (ε = 1). The analysis determined that a total sample size of 28 participants was needed, with 14 participants per group.

### 2.3. Randomization and Blinding

The participants were randomly assigned to either the experimental (SSE training) or control groups. To enrol individuals (1:1), the Research Randomizer software (version 4.0, Geoffrey C. Urbaniak and Scott Plous, Middletown, CT, USA) generated a randomization sequence [67]. The method was carried out by a member of the research team who was not actively involved in the trial’s clinical aspects. The group assignment was hidden in a password-protected computer file. Participants were aware of their group assignment, but outcome assessors and data analysts would be unaware of it.

### 2.4. Participants

A total of 44 healthy women (n = 24 experimental group with SSE; n = 20 control group) from a convenience sample participated in this study. Participants were recruited via the Exercise Looks After You programme [68] through face-to-face and phone interviews. All recruited participants fulfilled the following inclusion criteria: (a) were 65 years or older; (b) did not have or suffer from any ailment or disability that prevented normal physical activity; and (c) read, understood and signed the written informed consent form in line with the updated Helsinki declaration. Furthermore, subjects were also excluded if they had a physician-diagnosed neurodegenerative disease or scored less than 18 on the Montreal Cognitive Assessment (MoCA). The University Research Ethics Committee approved all methods (permission number: 92/2022), and all subjects provided verbal and written informed consent to participate in this study.

### 2.5. Procedures

The intervention lasted four months, which consisted of training from the beginning to the third month and a fourth month of follow-up without training. Evaluation lasted two days. Before beginning the measures, the participants were given instructions and considerations for the proper performance of the various fitness tests. On the first day, participants’ age, height (SECA 225, SECA, Hamburg, Germany) and body mass index (BMI) (TANITA MC-780 MA, Tanita Corp., Tokyo, Japan) were assessed. Subsequently, EEG was conducted and venous blood samples were collected by a health professional [69] according to the recommendations of the European Federation of Clinical Chemistry and Pre-Analytical Phase Working Group and the Latin American Pre-Analytical Phase Working Group [70]. After the EEG and venous blood measurements, participants completed the MoCA [71], International Physical Activity Questionnaire (IPAQ) [72], the 21-item Fall Risk Index [73,74] and Falls Efficacy Scale-International (FES-I) [75,76,77]. All the evaluation in the first day took place in the Faculty of Sport Sciences (Cáceres, Spain) in a humidity- and temperature-controlled laboratory. On the second day, the following physical tests were measured: the dynamic balance test Four-Square Step Test (FSST) [78]; the Imagined Time Up and Go (TUGI), Time Up and Go (TUG) and Time Up and Go Dual (TUGD) agility tests [79]; and the Brisk Walking (BW) and Brisk Walking Dual (BWD) tests [80] were used for gait speed. Lower limb strength was measured by the 30 s Sit-to-Stand (30S), 30 s Sit-to-Stand Imagined (30SI) and 30 s Sit-to-Stand Dual (30SD) tests [81]. Aerobic capacity was measured using the 6 min walk test [82]. All the evaluation in the second day took place in the park, where the subjects walked as usual. The arrangement of these tests was determined to avoid local weariness in the lower extremities.

### 2.6. Instruments, Processing and Outcomes

#### 2.6.1. Primary Outcome

##### Dynamic Balance

The FSST was used to assess mobility, balance and agility in older adults. It consists of walking across four markers arranged in a square in different directions (forward, backward, left and right) in as short a time as possible. The test measures speed and control in the transitions between the squares, making it possible to identify deficits in dynamic stability and motor coordination. The FSST was the main variable used in this study because it is the most specific in relation to the type of intervention carried out by the experimental group, which focused on improving the agility and control of movements. This test has been shown to be valid and reliable for predicting fall risk and assessing the effectiveness of balance interventions, and is accessible for use in clinical and community settings (ICC = 0.98) [78].

#### 2.6.2. Secondary Outcomes

##### Agility

The TUG, TUGD and TUGI tests were used. The participant stood up from a chair with a backrest, walked 3 m at their usual speed, made a turn and returned to the starting point to sit down again. The total time was measured with a stopwatch. Three attempts were made and the average time of these was used as the final score [79]. The TUGD test assesses an individual’s ability to perform cognitive tasks simultaneously while performing a motor activity. The TUGD test requires the participant to perform the same protocol as the TUG test, but with the addition of a cognitive task, such as counting backwards, while walking. This test provides information about the interaction between motor and cognitive performance. The TUGI test assessed the ability of individuals to imagine performing the test without physically performing the movements. This test requires the participant to imagine getting up from a chair, walking a given distance, turning around and returning, without performing the action. The TUGI test allowed the assessment of cognitive processes related to planning and anticipatory motor execution.

##### Gait Speed

The BW and BWD tests were used to assess gait speed under normal and dual conditions. The BW and BWD tests determine the time required to walk 30 m as quickly and safely as possible (ICC = 0.93) [80]. Two trials with one minute rest were performed. The best of the results was used for analysis. The BWD test is a variant that involves a cognitive task during the walk, in this case a countdown. This test assesses the ability to perform motor and cognitive tasks simultaneously, which is crucial for detecting deficits in executive function and the integration of complex tasks.

##### Lower Limb Strength

The 30S, 30SD and 30SI tests were used to measure lower limb strength. These tests evaluate the total number of times that a participant stands up completely from a seated position with a straight back and feet parallel to the floor, not including arm thrust, in 30 s (R = 0.89, CI = 0.79–0.93) [81]. The 30SD test combined the original test with a simultaneous cognitive task, such as performing a simple countdown while transitioning from sitting to standing. The 30SI test was a cognitive variant of the test in which the participant was asked to imagine the action of getting up from a chair without physically performing it.

##### Aerobic Capacity

The 6 min walk test was performed to assess aerobic capacity. This test determines the maximum distance that each participant can walk for 6 min around a 45.7 m rectangle (R = 0.94, CI = 0.90–0.96) [81].

##### Fall Risk

The 21-item Fall Risk Index (FRI-21) was used. It is a questionnaire with 21 items. Each item is assigned a rating of 1 (risk) or 0 (no risk), and the total number of elements ranges from 0 (minimum risk of falls) to 21 (severe risk). Elevated scores suggest a greater chance of falling. A cut-off points of 9–10 on the FRI-21 is beneficial for early identification of fall risk (sensitivity 0.65) [73,74].

##### Fear of Falling

The Falls Efficacy Scale-International (FES-I) was used to assess fear of falling. It is a questionnaire developed and authorised by the Prevention of Falls Network Europe. It has become a measure with great accuracy and authenticity for evaluating fear of falling (ICC = 0.96) in many societies and languages [75,76]. The first questionnaire had 16 items and was graded on a 4-point scale (1 = slightly anxious to 4 = highly worried). As a result, the best possible value was 16, while the worst was 64.

##### Electroencephalography (EEG)

According to the International 10–20 system, 19 EEG scalp locations were recorded using an Enobio device (Neuroelectrics, Cambridge, MA, USA) (1) with the NIC.1 software (Neuroelectrics, Cambridge, MA, USA). Frontal (Fz, Fp1, Fp2, F3, F4, F7, and F8), central (Cz, C3 and C4), temporal (T3, T4, T5, and T6), parietal (Pz, P3 and P4) and occipital (O1 and O2) locations were measured.

Two electrodes were placed in the earlobes as references. Impedance was kept below 10 KΩ during the visual stimulus. EEG was recorded at 500 Hz. The EEGlab toolbox (version 2021, executed in MatLab version R2019a) (2) was employed to pre-process data. Line noise was removed using a 1 Hz high-pass filter. Artefact Subspace Reconstruction (ASR) was used to reject bad channels and correct continuous data. Bad channels were interpolated, and data were re-referenced to average. Independent component analysis (ICA) was conducted. Single equivalent current dipoles were estimated and symmetrically constrained bilateral dipoles were searched. Independent Components (ICs) whose dipoles’ residual variance was larger than 15% were removed, as were those with dipoles located outside the brain. The EEGlab toolbox (MatLab) was used to compute and extract power spectral densities at different frequency bands: theta (4–7 Hz), alpha (8–12 Hz) and beta (13–30 Hz). Plot-averaged topography maps were extracted over the frequency range for baseline, three months and six months of intervention.

##### Blood Collection and Western Blotting for SMP30

Blood was collected by direct venipuncture into the antecubital vein using a 21-gauge butterfly needle (BD Vacutainer Safety-Lok™, BD Biosciences, Ryde, Australia). Blood samples were immediately mixed with citric acid dextrose (ACD) (1/6) to avoid blood clots due to subsequent manipulation. Blood was delivered the following hour to the laboratory, under refrigerated conditions. Upon arrival, samples were immediately centrifuged at 750× *g* for 5 min and, following this, were supplemented with aspirase (100 U/mL) and aspirin (100 µM) to prevent platelet activation and, therefore, the subsequent secretion of platelet granules that may have interfered with the biochemical determination. Then, the plasma containing the platelets was centrifuged again at 350× *g* for 20 min to isolate the plasma fraction free of platelets and, finally, 250 µL per plasma sample was collected and treated as described below. Samples were immediately stored at −80 °C for future analysis.

Plasma samples were thawed, and proteins were immediately denatured under reducing conditions by mixing with an equal volume of Laemmli buffer (2×, 10% DTT). Protein samples were heated to 90 °C for 5 min and were immediately frozen to allow final protein denaturalization. Protein samples were stored at −80 °C until required for analysis of SMP30 in all isolated samples simultaneously. Samples were pooled and loaded on the same gel by intercalating pre-test, post-test and follow-up samples. Protein isolation was performed using 10% sodium dodecyl sulphate–polyacrylamide gel electrophoresis (SDS-PAGE). Subsequently, Western blotting was performed by electro-transferring the proteins to nitrocellulose membranes; the membranes were then incubated for 1 h at room temperature with the specific anti-SMP30 antibody diluted 1:1000 in 0.1% Tris-buffered saline and Tween^®^ 20 detergent (TBST). After using the appropriate HRP-conjugated secondary antibody (1:10,000 for 1 h), the membranes were exposed to SuperSignal solution and optic densitometry was performed and analysed using the ChemiDoc Imaging System (Bio-Rad^®^, Madid, Spain). Membranes were reprobed with anti-actin antibody (1:1000 for 1 h) to ensure that similar amounts of protein were loaded on each gel line.

### 2.7. Exercise Interventions in SSE Group

This group trained twice a week for three months. The training took place on a 200 × 100 cm mat divided into 40 squares of 25 × 25 cm. The programme consisted of 200 movement patterns divided into levels of increasing difficulty: beginner, intermediate and advanced. During the sessions, participants learned and performed different step patterns, starting with basic movements and progressing to more complex patterns. The structure of the programme allowed for a gradual progression, increasing in difficulty over time, as described in Table 1 [83].

Table 2 describes the structure of an SSE training session in the experimental group. Before each session, the instructor explained the guidelines to be followed. First, a general and specific warm-up was performed. Then, the participants learned and performed the movement patterns programmed for that day, which varied between three and five depending on the level of difficulty. This was followed by a cool-down phase, which included stretching and a short relaxation phase to return the body to its initial state. At the end of each session, a final discussion was held in which the participants shared their opinions on the patterns they had learned and rated the perceived intensity of the effort they had made on a scale of 0 to 10, considering the tension and fatigue they had experienced. They were instructed to keep the perceived effort between 2 and 6. Participants in the control group continued with their usual lifestyle. At the end of the study, the SSE intervention was offered free of charge to participants in the control group.

### 2.8. Statistical Analysis

Statistical procedures and computations were conducted using the new “3rd generation” statistical spreadsheet Jamovi (version 2.5, Sydney, Australia) [84]. Data were presented as mean and standard deviation (SD). For the SMP30 variable, the baseline value was standardised to 100% expression to allow calculation of percentage changes from baseline and follow-up values. Data normality and homogeneity were checked by conducting Shapiro–Wilk and Levene tests, respectively. Then, inferential tests were conducted. A two-way repeated-measures analysis of variance (ANOVA) was performed to examine the interaction between two factors: the group (SSE and Control) and the three time points (pre-test, post-test and follow-up) for all dependent variables. Significant differences were set at *p* ≤ 0.05.

EEGLAB study design was used to compare the electrophysiological response at baseline, after three months of intervention and at the follow-up. Thus, an EEGLAB study design (2 × 3) was configured to compare the brain dynamics in the three moments. Non-parametric analysis (permutation analysis) was computed. To control the Type I error, the false discovery rate correction (FDR) was applied.

## 3. Results

In the present study, all participants assigned to the SSE group attended at least 85% of the scheduled sessions of the training protocol. This level of compliance ensures adequate exposure to the intervention programme, which is fundamental to the validity of the results obtained for the variables analysed. The results showed no significant differences between the two groups in any of the characteristics assessed at baseline (see Table 3), as well as in the EEG and SMP30 variables.

The repeated-measures ANOVA outcomes showed group x time interactions in the FSST (*p* = 0.001; F = 7.47), BW test (*p* < 0.001; F = 13.61), BWD test (*p* = 0.002; F = 6.98), 6 min walk test (*p* = 0.003; F = 6.26), FES-I (*p* = 0.004; F = 5.97), and FRI-21 (*p* = 0.002; F = 6.71). However, no interactions were observed for the TUG, dual-task TUG, imagined TUG, 30S, 30SI, 30SD, EEG and SMP30 variables (*p* > 0.05).

### 3.1. Dynamic Balance and Agility

**Figure 1:**
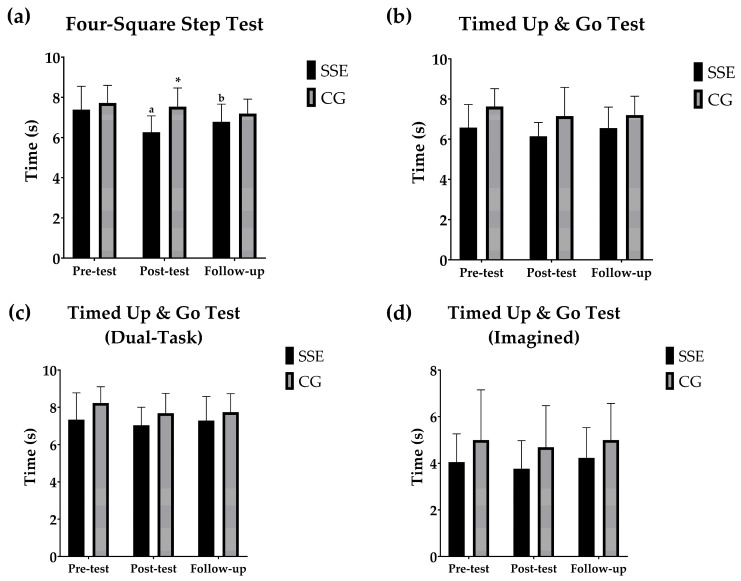
(**a**) Evolution of the mean and standard deviation of the seconds in the Four-Square Step test. (**b**) Changes in the mean and standard deviation of the seconds in the Timed Up and Go Test. (**c**) Development of the mean and standard deviation of the seconds in the Timed Up and Go Test (Dual-Task). (**d**) Variation in the mean and standard deviation of the seconds in the Timed Up and Go Test (Imagined). Pairwise within- and between-group comparisons between the different moments for the balance tests. ^a^ Within-group significant differences between the different time points compared to pre-test; ^b^ within-group significant differences between the different time points compared to post-test; and * between-group significant differences at the same time point.

Figure 1 shows the pairwise within- and between-group comparisons between the different moments for the balance tests. Significant differences were observed between pre-test and post-test values for the FSST (*p* < 0.001) in the SSE group, whereas no differences were found in the control group (*p* > 0.05). Similar results were reported when comparing post-test with follow-up, where the SSE group showed significant differences in their FSST times (*p* = 0.035), in contrast to the control group, which reported no significant changes (*p* > 0.05). However, no differences were found between pre-test and follow-up in any group (*p* > 0.05). Furthermore, no differences were observed in any group for the TUG, TUGD and TUGI tests (*p* > 0.05). Between the groups, the SSE group showed significantly better performance in the FSST at the post-test measurement compared to the control group (*p* < 0.001), indicating that the SSE group took significantly less time to complete the test. There were no significant differences between the groups at any of the other assessment points for the other variables.

### 3.2. Gait Speed and Aerobic Capacitiy

**Figure 2:**
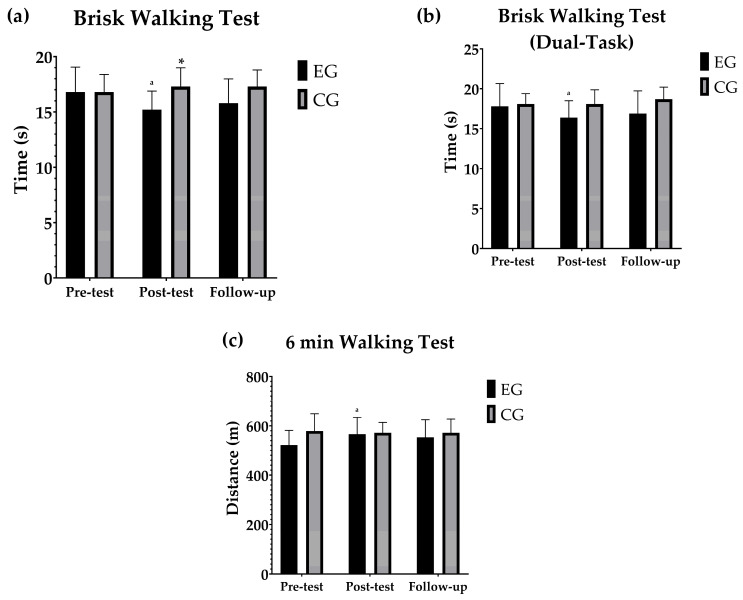
(**a**) Evolution of the mean and standard deviation of the seconds in the Brisk Walking Test. (**b**) Changes in the mean and standard deviation of the seconds in the Brisk Walking Test (Dual task). (**c**) Development of the mean and standard deviation of the distances in the 6 Min Walking Test. Pairwise within- and between-group comparisons between the different moments for the aerobic capacity and gait speed tests. ^a^ indicates the within-group significant differences between the different time points compared to pre-test, and * denotes significant between-group differences at the same time point.

Figure 2 shows the pairwise within- and between- group comparisons between the different moments for the gait speed and aerobic capacity tests. Significant differences were observed between the pre-test and post-test values for the BW (*p* < 0.001), BWD (*p* = 0.003) and 6 min walk (*p* = 0.002) tests in the SSE group, whereas no differences were found in the CG (*p* > 0.05). However, no differences were found between the pre-test and follow-up values or between the post-test and follow-up values in any group (*p* > 0.05). In the between-group comparisons, the SSE group showed significantly better performance in the BW test at the post-test time point compared to the control group (*p* = 0.013), indicating that the SSE group took significantly less time to complete the test. There were no significant differences between the groups at any of the other assessment points for the other variables.

### 3.3. Lower Limb Strength

**Figure 3:**
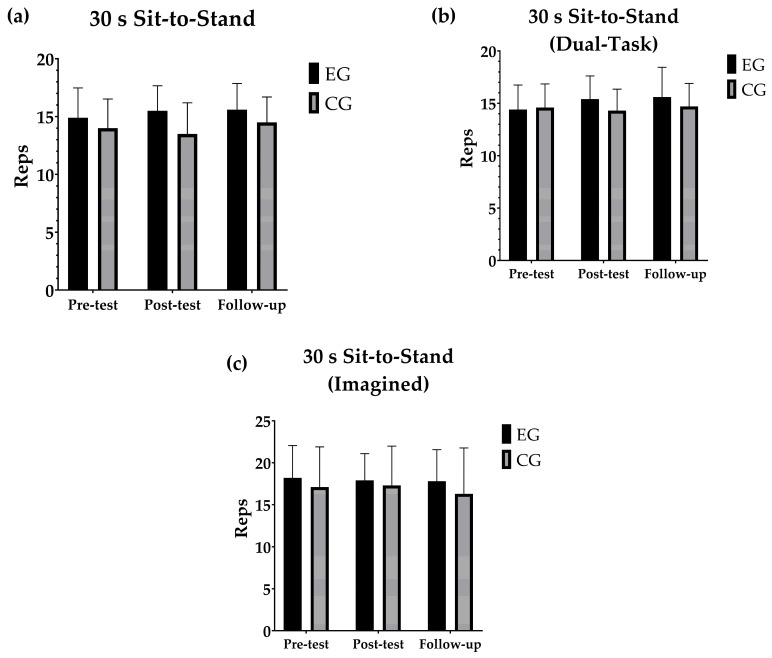
(**a**) Evolution of the mean and standard deviation of the number of repetitions in the 30 s Sit-to-Stand test. (**b**) Changes in the mean and standard deviation of the number of repetitions in the 30 s Sit-to-Stand test (Dual task). (**c**) Development of the mean and standard deviation of the number of repetitions in the 30-s Sit-to-Stand test (Imagined). Pairwise comparisons within and between groups across different time points for the lower limb strength tests.

Similarly, for the lower limb strength tests, the pairwise within- and between-group comparisons between the different time points are shown in Figure 3. No significant differences were observed between the different time points for SSE group (*p* > 0.05), nor for CG (*p* > 0.05). There were no significant differences between the groups at any of the assessment points.

### 3.4. Fall Risk and Fear of Falling

**Figure 4:**
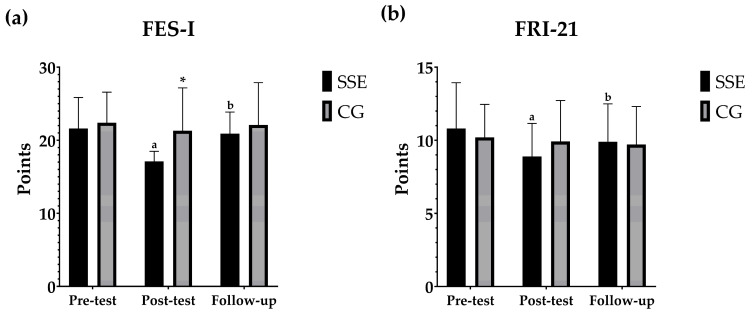
(**a**): Evolution of scoring on the FES-I scale. (**b**): Development of FRI-21 scale scores. Pairwise within- and between-group comparisons between the different moments for the FES-I and FRI-21 questionnaires. ^a^ indicates within-group significant differences between the different time points compared to pre-test; ^b^ indicates within-group significant differences between the different time points compared to post-test; and * indicates between-group significant differences at the same time point.

Similarly, Figure 4 shows the pairwise within- and between-group comparisons between the different moments for the fall risk and fear of fall questionnaires. Significant differences were observed between the pre-test and post-test results for the FES-I (*p* < 0.001) and FRI-21 (*p* < 0.001) in the SSE group, while no differences were found in the control group (*p* > 0.05). Similar results were reported when comparing the post-test with the follow-up, where the SSE group showed significant differences in FES-I (*p* < 0.001) and FRI-21 (*p* = 0.014) scores, in contrast to the control group which showed no significant changes (*p* > 0.05). Finally, no differences were found between pre-test and follow-up values in any group (*p* > 0.05). When comparing the groups, significant differences were observed in the FES-I in favour of the SSE group, who achieved a lower post-test score than the control group (*p* = 0.048). There were no significant differences between the groups on any of the other scores for either the FES-I or the FRI-21.

### 3.5. Cognitive Function

**Figure 5:**
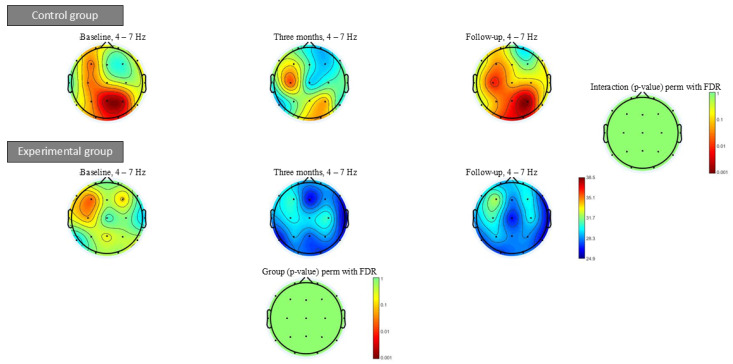
Theta power spectrum (4–7 Hz) topographic maps at baseline, after three months of intervention and at follow-up after intervention. Significant differences were not found in any frequency bands after intervention.

Figure 5 shows the theta power spectrum at baseline, after three months of intervention and at follow-up. Within- or between-group significant differences were not found in any of the power spectrum bands analysed.

**Figure 6:**
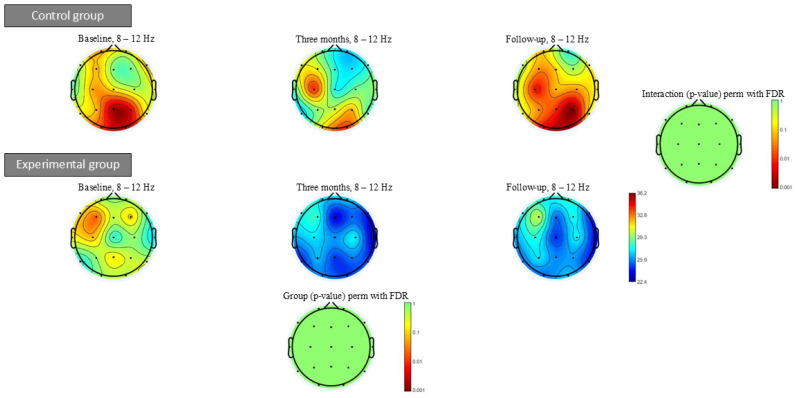
Alpha power spectrum (8–12 Hz) topographic maps at baseline, three months of intervention and follow-up. Significant differences were not found in any frequency bands after intervention.

Figure 6 shows the alpha power spectrum at baseline, after three months of intervention and at follow-up. Within- or between-group significant differences were not found in any of the power spectrum bands analysed.

**Figure 7:**
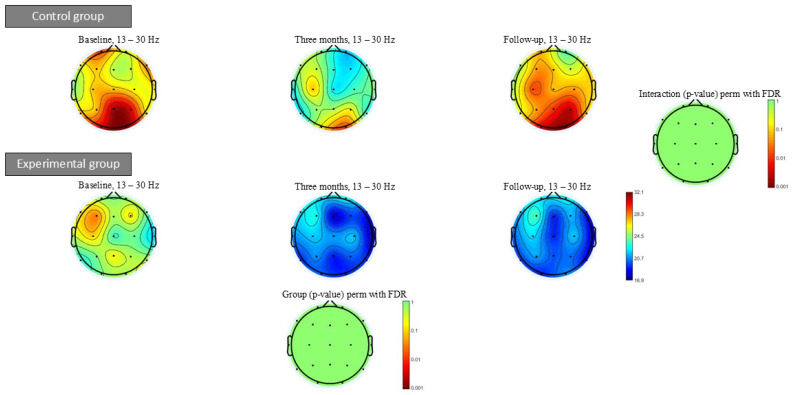
Beta power spectrum (13–30 Hz) topographic maps at baseline, three months of intervention and follow-up. Significant differences were not found in any frequency bands after intervention.

Figure 7 shows the beta power spectrum at baseline, after three months of intervention and at follow-up. Within- or between-group significant differences were not found in any of the power spectrum bands analysed.

### 3.6. Plasma Content of SMP30

**Figure 8:**
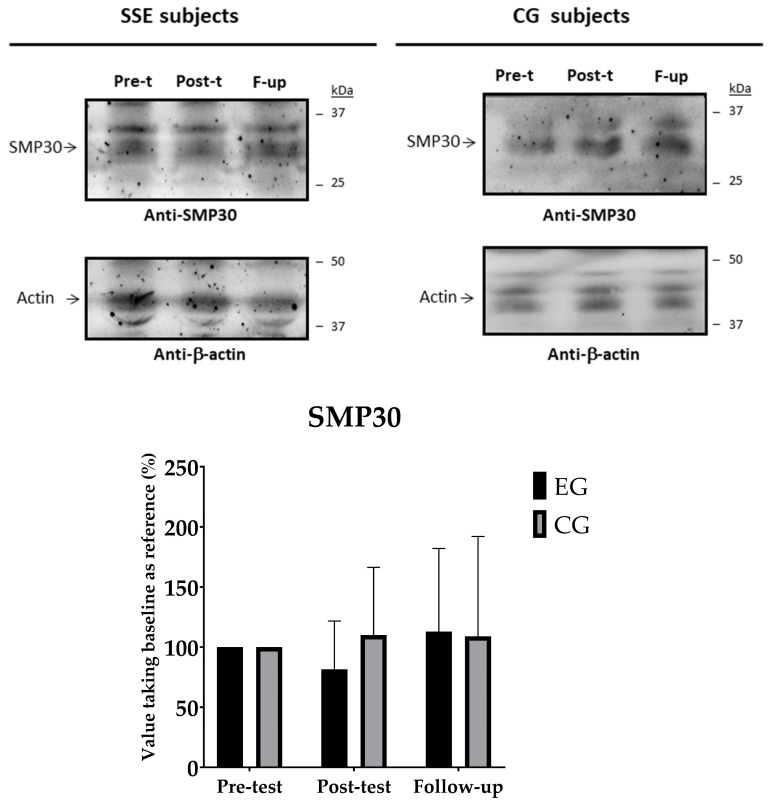
Plasma SMP30 expression. Paired comparisons within and between groups between different time points.

Figure 8 illustrates the pairwise comparisons within and between groups at different time points for SMP30. Baseline values were standardised to 100% and the post-test and follow-up measurements were expressed as percentage changes to reflect increases or decreases in SMP30 levels. No significant changes were observed at any time point in either the SSE group (*p* > 0.05) or the control group (*p* > 0.05). In addition, no significant differences were observed between the groups at any time point.

## 4. Discussion

The main objective of this study was to analyse and evaluate the effect of an SSE training programme on physical and cognitive function and plasma SMP30 levels for the prevention of falls in older adults. The main results of the study showed that the experimental group achieved significant improvements over the control group in the FSST, BW and FES-I tests following the training programme. No significant differences were observed in other physical function variables, EEG activity and plasma SMP30 expression levels.

### 4.1. Physical Function

In terms of physical function outcomes, SSE showed improvements in functional balance and gait speed in the older population as measured by the FSST [66] and BW test [80], respectively. The results revealed significant improvements in the SSE group compared to the control group after the training period, suggesting that the SSE programme may be an effective intervention to improve stability and gait speed, thus potentially reducing the risk of falls. These results are consistent with previous studies showing that sequential pattern exercise programmes, such as SSE, have a beneficial effect on improving postural control and balance in older adults [62]. These benefits may be due to the fact that these steps, which are performed in different directions, create a narrower base of support that challenges balance [85]. In addition, other studies suggest that the long-term practice of these exercises could alter motor and cognitive levels in the process of correcting the disturbances of these directional changes [86]. Other authors have reported that both postural control and dynamic balance, together with agility, are fundamental elements to avoid the risk of falls in the older-adult population, and the FSST is one of the tests that provides improvements in these elements [87,88]. However, further studies are needed to confirm the results obtained in the FSST, as its similarity to the tasks performed in the SSE could influence the results, leading to a possible task specificity effect. On the other hand, the improvement observed in the FSST for the SSE group was not maintained at follow-up (*p* = 0.035), suggesting that the benefits of the programme diminished over time, probably due to the lack of a continuous exercise stimulus. This is consistent with Teixeira et al., 2004 [89], who found that only one month of no exercise was sufficient to worsen parameters related to functional capacity in older people, including gait and balance. These findings highlight the importance of sustained physical activity, particularly in programmes that develop muscle strength and the ability to change direction, to reduce the risk and fear of falling [90].

The improvement in the SSE group compared with the control group on the BW test is of great clinical importance, given its close relationship with daily functioning and independence in older adults [91]. Our results are consistent with those of Shigematsu et al. (2006) [60], who reported significant improvements over the control group in the “walking around two cones” test in a group that performed SSE once a week for 24 weeks. Similarly, Shigematsu et al. (2008) [61] found significant improvements in gait speed tests in the SSE group compared to a walking group (*p* = 0.03) after 12 weeks of twice-weekly training. However, when SSE programmes were compared with strength training, both showed significant improvements in gait speed tests but neither proved superior after 12 weeks of training three times a week [62]. This body of evidence highlights that although SSE is an effective tool for improving gait speed and certain aspects of mobility, its inclusion in more comprehensive training programmes could improve outcomes across a wider range of physical abilities, thereby optimising its impact on fall prevention.

Although the SSE group improved significantly after the intervention in the BWD and 6 min walk tests (*p* < 0.05), no significant changes were observed in the comparisons between the groups in the other physical function variables (TUG, TUGD, TUGI, BWD, 30S, 30SD, 30SI and 6 min walk) (*p* > 0.05). This finding is significant as it suggests that although the SSE programme is effective in improving dynamic balance as measured by the FSST, it does not appear to have a significant effect on other aspects of functional mobility, as assessed by the TUG test. Contrary to expectations, the results of the TUG and TUG dual-task tests showed better balance and gait scores, or even better times, in the dual-task variant than in the normal variant [92,93], although similar results were not found in this study with the SSE protocol. A possible explanation could be that SSE focuses mainly on the coordination and sequencing of multidirectional steps, which may not be sufficient to influence performance in mobility tasks that require greater speed or cognitive integration, such as the TUG dual-task test. In addition, research has found evidence of cognitive enhancement, suggesting that hybrid SSE, as a cognitive–physical exercise with cues that involve cognitive actions, such as remembering and executing the patterns displayed on the monitor, causes older adults to consciously modify their habitual way of walking, directly affecting their gait performance and dynamic balance [94].

Similarly, no significant improvements in lower limb strength were observed after the SSE intervention. Shigematsu et al. (2008) [61] found no significant increases in lower limb strength when comparing an SSE group with a walking group after 12 weeks of twice-weekly training, likely due to the emphasis of SSE on coordination, balance and gait rather than strength. In contrast, Cha et al. (2022) [95] reported significant improvements in strength (*p* < 0.05) after 12 weeks of SSE in participants over 60 years of age. Differences in sample size (Cha et al. included 10 participants per group versus 20 in our study) or baseline physical activity levels may explain these discrepancies. It is possible that the initial physical capacity of participants in some studies was insufficient to achieve strength gains.

### 4.2. Cognitive Function

The evaluation of EEG activity showed no significant differences between the groups in any of the frequency bands. Although SSE improved physical aspects related to balance, the lack of significant changes in brain activity could be due to several factors. The nature of the SSE, which focuses on relatively simple motor patterns, may not have been stimulating enough to produce detectable brainwave changes in older people. However, a resistance training programme of 15 repetitions at 65% of 1 RM using elastic bands for 12 weeks was shown to significantly improve relative theta power at P3 (*p* < 0.05) and P4 (*p* < 0.05), suggesting improvements in cognitive function in older people [96]. Another study showed that EEG spectral power in older people who exercised regularly correlated with exercise intensity (*p* < 0.001), but there was no correlation in older people who exercised occasionally [97]. Similarly, lower limb strength training combined with whole-body vibration was shown to significantly improve signal power in the C4, F7, P3 and P4 electrodes (*p* < 0.05) in people over 60 years of age, using a vibration frequency of 30 Hz for 20 s [98]. These findings suggest that exercise intensity may be a relevant factor in modifying EEG activity, but further research is needed to support this.

In addition, EEG analysis was performed at rest, which may limit its ability to capture brain adaptations that may be more evident during the performance of dynamic or cognitive tasks [59]. Factors such as sample size and inter-individual variability may also have contributed to these results, as brain responses to exercise can vary considerably between participants due to factors such as age, physical condition or previous exercise experience [99]. This highlights the need for future studies that conduct EEG during exercise or include cognitive tasks to further investigate the effects of SSE on brain activity.

### 4.3. Plasma Content of SMP30

Finally, as expected because of the low range of changes in muscle parameters like lower limb strength, our study showed no significant changes in plasma SMP30 levels. To the best of our knowledge, no previous studies have used SSE training protocols to assess plasma SMP30 expressions. One study examined the effects of a multi-component training programme (strength, endurance, balance, coordination and flexibility) for six months on plasma SMP30 levels in frail older people [39]. The results showed no significant changes in plasma SMP30 levels at the time points tested. However, the authors pointed out that comparisons were only made between the third and sixth months, as no baseline samples were taken at the start of the intervention. They pointed out that it would have been ideal to measure SMP30 levels from baseline to three months after training. According to the authors, it was possible that SMP30 levels were higher after the first three months, while the ability to increase them between months three and six may be limited. They also suggested that the multi-component training features may not have been effective enough to affect SMP30 expressions. In contrast, the same research group analysed the effect of 12 weeks of vibration training in postmenopausal women on this protein [38]. The results showed a significant increase in plasma SMP30 (*p* = 0.004), but it should be noted that this study was not compared to a control group. According to the authors, this increase could be associated with a 4.4% reduction in body fat. This is based on previous evidence suggesting that SMP30 is involved in hepatic lipid regulation [100]. Another possible explanation for the increase in SMP30 is the prolonged exposure to the vibration training programme. This type of training may alter intracellular and circulating levels of SMP30. The researchers observed discrepancies in bone mineral content associated with this training programme. Such changes may be related to the regulatory role of SMP30 in osteoblastic and osteoclastic cells [101]. Given the lack of clear consensus and the limited number of studies, further research is needed to clarify the effects of exercise programmes on plasma SMP30 levels.

### 4.4. Limitations

The strength of this study lies in the combination of electroencephalographic measures, plasma levels of SMP30, and objective physical tests that allow the assessment of gait, fall risk and cognitive activity. However, this study has several limitations that need to be considered. First, the relatively small sample size (44 participants) may have limited its statistical power, making it difficult to detect subtle changes in the variables analysed. Also, the inclusion criterion based on a MoCA score of 18 or more, which is lower than the generally accepted cut-off of 26, may have allowed the inclusion of participants with possible mild cognitive impairment. This should be considered when interpreting the results, as our sample may not be exclusively representative of older people without cognitive impairment. In addition, the EEG analysis was performed at rest, which provides a controlled environment but may have missed possible exercise-induced changes in brain activity during dynamic tasks. Also, the lack of measurement of cognitive effects during exercise and limited control for confounding factors such as diet or stress may have influenced the results. In addition, the duration of the intervention was relatively short (three months), which may have influenced the lack of changes observed in muscle strength and SMP30 expression. It is possible that a longer intervention period may be required to observe significant changes in these markers. The potential of advanced technologies, such as inertial sensors, 3D analysis and force platforms, to provide more accurate assessments of changes in gait patterns and lower limb strength was not used. The absence of a control group combining SSE with resistance exercise precluded an analysis of whether a more comprehensive intervention could have yielded superior outcomes. Furthermore, the participants were already engaged in physical activity prior to the study, which may have influenced the extent of improvements observed and made it challenging to discern changes in specific biomarkers. Finally, the homogeneity of the sample, which consisted exclusively of active older women, limits the generalisability of the findings to other populations, such as men or people with chronic diseases.

### 4.5. Practical Applications

SSE has important practical applications, as it is an accessible, low-cost intervention that can be adapted to different settings, such as community centres and homes. Its simplicity and adaptability make it feasible for older populations and improve long-term adherence, which is essential for maintaining the benefits of exercise. The results of this study, showing significant improvements in dynamic balance, gait speed and reduced fear of falling, have clear implications for preventing serious injury in older people, reducing the need for hospitalisation and associated costs. In addition, by reducing the fear of falling and improving stability, SSE helps older people maintain their functional independence for longer, improving their quality of life and reducing the burden on healthcare systems. Given that falls are a major cause of disability in this population, the use of SSE in prevention programmes could be a key tool in promoting healthy ageing.

## 5. Conclusions

The results of this study suggest that an SSE programme is effective in improving functional balance, gait speed and reducing the fear of falling in older people. However, no significant effects were observed in other areas of physical function, such as agility, lower limb strength and aerobic capacity, and improvements in functional balance diminished at follow-up after the end of the programme, highlighting the need for continued practice to maintain long-term benefits. In addition, this study found no significant changes in plasma SMP30 levels or the cognitive parameters assessed. These findings highlight the need for further research to investigate the effects of SSE on different functional and biological domains in this population.

## Figures and Tables

**Table 1:** Square Stepping Exercise (SSE) progression for intervention.

Month	Weeks	Frequency (Days a Week)	Session Time (min)	Difficulty (Level)
1	1–2 3–4	2	60	Beginner 1 Beginner 2 Intermediate 1
2	5–6 7–8	2	60	Intermediate 2 Intermediate 3 Intermediate 4
3	9–10 11–12	2	60	Advanced 1 Advanced 2

**Table 2:** Usual composition of an SSE session.

Warm-Up (10 min)
Joint movement
**Main Part (40 min)**
Patch of patterns realised in prior session
Understanding and execution of SSE pattern 1
Understanding and execution of SSE pattern 2
Understanding and execution of SSE pattern 3
Understanding and execution of SSE pattern 4
**Cool-Down (10 min)**
Stretch
Rest
Final thought

**Table 3:** Descriptive characteristics of participants assigned to control or SSE groups.

Variables	Control Group (n = 24) Mean ± SD	SSE Group (n = 20) Mean ± SD	*p*-Value
Age (years)	68.8 ± 3.45	68.9 ± 4.17	0.722
BMI (kg)	26.0 ± 4.10	28.8 ± 5.24	0.134
Height (cm)	160.2 ± 7.82	156.2 ± 6.93	0.054
MoCA (points)	25.9 ± 3.46	27.4 ± 2.4	0.195
IPAQ (METs)	3816.0 ± 1654.29	3295.7 ± 796.15	0.406
FSST (s)	7.72 ± 0.877	7.39 ± 1.16	1.00
TUG (s)	7.63 ± 0.882	6.74 ± 1.03	0.054
TUGD (s)	8.23 ± 0.879	7.34 ± 1.44	0.238
TUGI (s)	5.00 ± 2.15	4.05 ± 1.21	1.00
BW (s)	16.8 ± 1.58	16.8 ± 2.25	1.00
BW Dual (s)	18.1 ± 1.29	17.8 ± 2.85	1.00
6 min walk test (m)	579 ± 69.6	522 ± 59.3	0.107
30S (rep)	14.0 ± 2.52	14.9 ± 2.58	1.00
30SI (rep)	17.1 ± 4.79	18.2 ± 3.85	1.00
30SD (rep)	14.6 ± 2.25	14.4 ± 2.35	1.00
FRI-21 (points)	10.2 ± 2.25	10.8 ± 3.14	1.00
FES-I (points)	22.4 ± 4.17	21.6 ± 4.24	1.00

30S: 30 s Sit-to-Stand; 30SD: 30 s Sit-to-Stand Dual; 30SI: 30 s Sit-to-Stand Imagined; BMI: Body Mass Index; cm: centimetres; BW: Brisk Walking; FES-I: Falls Efficacy Scale-International; FRI-21: 21-item Fall Risk Index; FSST: Four-Square Step Test; IPAQ: International Physical Activity Questionnaire; kg: kilograms; m: metres; METs: Metabolic Equivalents of Task; MoCA: Montreal Cognitive Assessment; rep: repetitions; s: seconds; SD: standard deviation; SSE: Square Step Exercise; TUG: Time Up and Go; TUGD: Time Up and Go Dual; TUGI: Imagined Time Up and Go.

## Data Availability

The average of the data from all subjects is included in the article.

## References

[1] Shetty P. (2012). Grey Matter: Ageing in Developing Countries. Lancet.

[2] Pasco J.A., Duque G. (2019). Age-Related Changes in Muscle and Bone. Osteosarcopenia: Bone, Muscle and Fat Interactions.

[3] Rezuş E., Burlui A., Cardoneanu A., Rezuş C., Codreanu C., Pârvu M., Rusu Zota G., Tamba B.I. (2020). Inactivity and Skeletal Muscle Metabolism: A Vicious Cycle in Old Age. Int. J. Mol. Sci..

[4] Friedman S.M. (2020). Lifestyle (Medicine) and Healthy Aging. Clin. Geriatr. Med..

[5] Accardi G., Aiello A., Caruso C., Candore G. (2021). Chapter 14—Ways to Become Old: Role of Lifestyle in Modulation of the Hallmarks of Aging. Human Aging.

[6] Xu Q., Ou X., Li J. (2022). The Risk of Falls among the Aging Population: A Systematic Review and Meta-Analysis. Front. Public Health.

[7] Grassi L., Caruso R., Da Ronch C., Härter M., Schulz H., Volkert J., Dehoust M., Sehner S., Suling A., Wegscheider K. (2020). Quality of Life, Level of Functioning, and Its Relationship with Mental and Physical Disorders in the Elderly: Results from the MentDis_ICF65+ Study. Health Qual. Life Outcomes.

[8] Cuevas-Trisan R. (2019). Balance Problems and Fall Risks in the Elderly. Clin. Geriatr. Med..

[9] Hausdorff J.M., Rios D.A., Edelberg H.K. (2001). Gait Variability and Fall Risk in Community-Living Older Adults: A 1-Year Prospective Study. Arch. Phys. Med. Rehabil..

[10] Tinetti M.E., Speechley M., Ginter S.F. (1988). Risk Factors for Falls among Elderly Persons Living in the Community. N. Engl. J. Med..

[11] Centers for Disease Control and Prevention Workplace Health Glossary|Tools & Resources|Workplace Health Promotion|CDC. https://www.cdc.gov/steadi/materials/building-clinical-strategies-to-steadi-your-older-patients_508_final.pptx.

[12] Galet C., Zhou Y., Eyck P.T., Romanowski K.S. (2018). Fall Injuries, Associated Deaths, and 30-Day Readmission for Subsequent Falls Are Increasing in the Elderly US Population: A Query of the WHO Mortality Database and National Readmission Database from 2010 to 2014. Clin. Epidemiol..

[13] Willett W.C., Koplan J.P., Nugent R., Dusenbury C., Puska P., Gaziano T.A., Jamison D.T., Breman J.G., Measham A.R., Alleyne G., Claeson M., Evans D.B., Jha P., Mills A., Musgrove P. (2006). Prevention of Chronic Disease by Means of Diet and Lifestyle Changes. Disease Control Priorities in Developing Countries.

[14] Berg W.P., Alessio H.M., Mills E.M., Tong C. (1997). Circumstances and Consequences of Falls in Independent Community-Dwelling Older Adults. Age Ageing.

[15] Ambrose A.F., Paul G., Hausdorff J.M. (2013). Risk Factors for Falls among Older Adults: A Review of the Literature. Maturitas.

[16] Taylor M.E., Ketels M.M., Delbaere K., Lord S.R., Mikolaizak A.S., Close J.C.T. (2012). Gait Impairment and Falls in Cognitively Impaired Older Adults: An Explanatory Model of Sensorimotor and Neuropsychological Mediators. Age Ageing.

[17] Rubenstein L.Z. (2006). Falls in Older People: Epidemiology, Risk Factors and Strategies for Prevention. Age Ageing.

[18] Papalia G.F., Papalia R., Diaz Balzani L.A., Torre G., Zampogna B., Vasta S., Fossati C., Alifano A.M., Denaro V. (2020). The Effects of Physical Exercise on Balance and Prevention of Falls in Older People: A Systematic Review and Meta-Analysis. J. Clin. Med..

[19] Langhammer B., Bergland A., Rydwik E. (2018). The Importance of Physical Activity Exercise among Older People. BioMed Res. Int..

[20] Moreland J.D., Richardson J.A., Goldsmith C.H., Clase C.M. (2004). Muscle Weakness and Falls in Older Adults: A Systematic Review and Meta-Analysis. J. Am. Geriatr. Soc..

[21] Shin S., Wuensche B. (2023). What Type of Exercises Should Older Adults Engage in to Improve Fall Efficacy and Physical Fitness Related to Falling?. J. Exerc. Rehabil..

[22] Arrieta H., Rezola-Pardo C., Gil S.M., Irazusta J., Rodriguez-Larrad A. (2018). Physical Training Maintains or Improves Gait Ability in Long-Term Nursing Home Residents: A Systematic Review of Randomized Controlled Trials. Maturitas.

[23] Bai X., Soh K.G., Omar Dev R.D., Talib O., Xiao W., Soh K.L., Ong S.L., Zhao C., Galeru O., Casaru C. (2022). Aerobic Exercise Combination Intervention to Improve Physical Performance Among the Elderly: A Systematic Review. Front. Physiol..

[24] Chen N., He X., Feng Y., Ainsworth B.E., Liu Y. (2021). Effects of Resistance Training in Healthy Older People with Sarcopenia: A Systematic Review and Meta-Analysis of Randomized Controlled Trials. Eur. Rev. Aging Phys. Act..

[25] Oesen S., Halper B., Hofmann M., Jandrasits W., Franzke B., Strasser E.-M., Graf A., Tschan H., Bachl N., Quittan M. (2015). Effects of Elastic Band Resistance Training and Nutritional Supplementation on Physical Performance of Institutionalised Elderly—A Randomized Controlled Trial. Exp. Gerontol..

[26] Sepúlveda M., Arauna D., García F., Albala C., Palomo I., Fuentes E. (2022). Frailty in Aging and the Search for the Optimal Biomarker: A Review. Biomedicines.

[27] Colucci-D’Amato L., Speranza L., Volpicelli F. (2020). Neurotrophic Factor BDNF, Physiological Functions and Therapeutic Potential in Depression, Neurodegeneration and Brain Cancer. Int. J. Mol. Sci..

[28] Álvarez-Sánchez N., Álvarez-Ríos A.I., Guerrero J.M., García-García F.J., Rodríguez-Mañas L., Cruz-Chamorro I., Lardone P.J., Carrillo-Vico A. (2020). Homocysteine and C-Reactive Protein Levels Are Associated With Frailty in Older Spaniards: The Toledo Study for Healthy Aging. J. Gerontol. A. Biol. Sci. Med. Sci..

[29] Ma L., Sha G., Zhang Y., Li Y. (2018). Elevated Serum IL-6 and Adiponectin Levels Are Associated with Frailty and Physical Function in Chinese Older Adults. Clin. Interv. Aging.

[30] Bermejo I., Carnicero J.A., Garcia F.J., Pérez-Baos S., Mateos M., Medina J.P., Mediero A., Rodríguez L., Largo R., Herrero-Beaumont G. (2022). Pos1446 Creatine Kinase Could Be a Marker of Chronic Inflammation-Induced Sarcopenia in Frail Patients. Ann. Rheum. Dis..

[31] Laurent M.R., Dupont J., Dejaeger M., Gielen E. (2019). Myostatin: A Powerful Biomarker for Sarcopenia and Frailty?. Gerontology.

[32] Pfeifer M., Begerow B., Minne H.W. (2002). Vitamin D and Muscle Function. Osteoporos. Int..

[33] Fujita T. (1999). Senescence Marker Protein-30 (SMP30): Structure and Biological Function. Biochem. Biophys. Res. Commun..

[34] Yamaguchi M., Mori S. (1988). Effects of Ca^2+^ and Zn^2+^ on 5’-Nucleotidase Activity in Rat Liver Plasma Membranes: Hepatic Calcium-Binding Protein (Regucalcin) Reverses the Ca^2+^ Effect. Chem. Pharm. Bull..

[35] Fujita T., Mandel J.-L., Shirasawa T., Hino O., Shirai T., Maruyama N. (1995). Isolation of cDNA Clone Encoding Human Homologue of Senescence Marker Protein-30 (SMP30) and Its Location on the X Chromosome. Biochim. Biophys. Acta BBA-Gene Struct. Expr..

[36] Mo Z., Zheng S., Lv Z., Zhuang Y., Lan X., Wang F., Lu X., Zhao Y., Zhou S. (2016). Senescence Marker Protein 30 (SMP30) Serves as a Potential Prognostic Indicator in Hepatocellular Carcinoma. Sci. Rep..

[37] Kondo Y., Inai Y., Sato Y., Handa S., Kubo S., Shimokado K., Goto S., Nishikimi M., Maruyama N., Ishigami A. (2006). Senescence Marker Protein 30 Functions as Gluconolactonase in L-Ascorbic Acid Biosynthesis, and Its Knockout Mice Are Prone to Scurvy. Proc. Natl. Acad. Sci. USA.

[38] Pérez-Gómez J., Adsuar J.C., García-Gordillo M.Á., Muñoz P., Romo L., Maynar M., Gusi N., PC R. (2020). Twelve Weeks of Whole Body Vibration Training Improve Regucalcin, Body Composition and Physical Fitness in Postmenopausal Women: A Pilot Study. Int. J. Environ. Res. Public Health.

[39] Pérez-Gómez J., Redondo P.C., Navarrete-Villanueva D., Lozano-Berges G., Ara I., González-Gross M., Casajus J.A., Vicente-Rodríguez G. (2021). New Evidence on Regucalcin, Body Composition, and Walking Ability Adaptations to Multicomponent Exercise Training in Functionally Limited and Frail Older Adults. Int. J. Environ. Res. Public Health.

[40] Raz N., Lindenberger U., Rodrigue K.M., Kennedy K.M., Head D., Williamson A., Dahle C., Gerstorf D., Acker J.D. (2005). Regional Brain Changes in Aging Healthy Adults: General Trends, Individual Differences and Modifiers. Cereb. Cortex.

[41] Resnick S.M., Pham D.L., Kraut M.A., Zonderman A.B., Davatzikos C. (2003). Longitudinal Magnetic Resonance Imaging Studies of Older Adults: A Shrinking Brain. J. Neurosci..

[42] Gunning-Dixon F.M., Brickman A.M., Cheng J.C., Alexopoulos G.S. (2009). Aging of Cerebral White Matter: A Review of MRI Findings. Int. J. Geriatr. Psychiatry.

[43] Babiloni C., Lizio R., Marzano N., Capotosto P., Soricelli A., Triggiani A.I., Cordone S., Gesualdo L., Del Percio C. (2016). Brain Neural Synchronization and Functional Coupling in Alzheimer’s Disease as Revealed by Resting State EEG Rhythms. Int. J. Psychophysiol..

[44] Rossini P.M., Rossi S., Babiloni C., Polich J. (2007). Clinical Neurophysiology of Aging Brain: From Normal Aging to Neurodegeneration. Prog. Neurobiol..

[45] de Bruin E.D., Schmidt A. (2010). Walking Behaviour of Healthy Elderly: Attention Should Be Paid. Behav. Brain Funct..

[46] Segev-Jacubovski O., Herman T., Yogev-Seligmann G., Mirelman A., Giladi N., Hausdorff J.M. (2011). The Interplay between Gait, Falls and Cognition: Can Cognitive Therapy Reduce Fall Risk?. Expert Rev. Neurother..

[47] Yogev G., Hausdorff J.M., Giladi N. (2008). The Role of Executive Function and Attention in Gait. Mov. Disord. Off. J. Mov. Disord. Soc..

[48] Holtzer R., Verghese J., Xue X., Lipton R.B. (2006). Cognitive Processes Related to Gait Velocity: Results from the Einstein Aging Study. Neuropsychology.

[49] Mirelman A., Herman T., Brozgol M., Dorfman M., Sprecher E., Schweiger A., Giladi N., Hausdorff J.M. (2012). Executive Function and Falls in Older Adults: New Findings from a Five-Year Prospective Study Link Fall Risk to Cognition. PLoS ONE.

[50] Delbaere K., Close J.C.T., Heim J., Sachdev P.S., Brodaty H., Slavin M.J., Kochan N.A., Lord S.R. (2010). A Multifactorial Approach to Understanding Fall Risk in Older People. J. Am. Geriatr. Soc..

[51] Pichierri G., Wolf P., Murer K., de Bruin E.D. (2011). Cognitive and Cognitive-Motor Interventions Affecting Physical Functioning: A Systematic Review. BMC Geriatr..

[52] Bamidis P.D., Vivas A.B., Styliadis C., Frantzidis C., Klados M., Schlee W., Siountas A., Papageorgiou S.G. (2014). A Review of Physical and Cognitive Interventions in Aging. Neurosci. Biobehav. Rev..

[53] Wright M.J., Karageorghis C.I., Nowicky A.V. (2020). Measuring Electrical Activity in the Brain during Exercise: A Review of Methods, Challenges, and Opportunities. Sport Exerc. Psychol. Rev..

[54] Teixeira E., Fonseca H., Diniz-Sousa F., Veras L., Boppre G., Oliveira J., Pinto D., Alves A.J., Barbosa A., Mendes R. (2021). Wearable Devices for Physical Activity and Healthcare Monitoring in Elderly People: A Critical Review. Geriatrics.

[55] Hosang L., Mouchlianitis E., Guérin S.M.R., Karageorghis C.I. (2022). Effects of Exercise on Electroencephalography-Recorded Neural Oscillations: A Systematic Review. Int. Rev. Sport Exerc. Psychol..

[56] Joundi R.A., Jenkinson N., Brittain J.-S., Aziz T.Z., Brown P. (2012). Driving Oscillatory Activity in the Human Cortex Enhances Motor Performance. Curr. Biol..

[57] De Sanctis P., Butler J.S., Malcolm B.R., Foxe J.J. (2014). Recalibration of Inhibitory Control Systems during Walking-Related Dual-Task Interference: A Mobile Brain-Body Imaging (MOBI) Study. NeuroImage.

[58] Pedroso R.V., Lima-Silva A.E., Tarachuque P.E., Fraga F.J., Stein A.M. (2021). Efficacy of Physical Exercise on Cortical Activity Modulation in Mild Cognitive Impairment: A Systematic Review. Arch. Phys. Med. Rehabil..

[59] Gramkow M.H., Hasselbalch S.G., Waldemar G., Frederiksen K.S. (2020). Resting State EEG in Exercise Intervention Studies: A Systematic Review of Effects and Methods. Front. Hum. Neurosci..

[60] Shigematsu R., Okura T. (2006). A Novel Exercise for Improving Lower-Extremity Functional Fitness in the Elderly. Aging Clin. Exp. Res..

[61] Shigematsu R., Okura T., Nakagaichi M., Tanaka K., Sakai T., Kitazumi S., Rantanen T. (2008). Square-Stepping Exercise and Fall Risk Factors in Older Adults: A Single-Blind, Randomized Controlled Trial. J Gerontol.

[62] Shigematsu R., Okura T., Sakai T., Rantanen T. (2008). Square-Stepping Exercise versus Strength and Balance Training for Fall Risk Factors. Aging Clin. Exp. Res..

[63] Sadeghian F., Zolaktaf V., Shigematsu R. (2023). A Comparison between Effects of Square-Stepping Exercise and Tai Chi Chuan on Functional Fitness and Fear of Falling in Older Women. Aging Clin. Exp. Res..

[64] Turner L., Shamseer L., Altman D.G., Weeks L., Peters J., Kober T., Dias S., Schulz K.F., Plint A.C., Moher D. (2012). Consolidated Standards of Reporting Trials (CONSORT) and the Completeness of Reporting of Randomised Controlled Trials (RCTs) Published in Medical Journals. Cochrane Database Syst. Rev..

[65] Erdfelder E., Faul F., Buchner A. (1996). GPOWER: A General Power Analysis Program. Behav. Res. Methods Instrum. Comput..

[66] Moore M., Barker K. (2017). The Validity and Reliability of the Four Square Step Test in Different Adult Populations: A Systematic Review. Syst. Rev..

[67] Urbaniak G.C., Plous S. (2013). Research Randomizer 2013 (Version 4.0) [Computer Software]. http://www.randomizer.org/.

[68] Gusi N., Herrera E., Quesada F., Cebrian C., Juan C.C. (2008). Exercise Looks after You: From Research to Practice in Elderly. J. Aging Phys. Act..

[69] Ha U., Lee Y., Kim H., Roh T., Bae J., Kim C., Yoo H.-J. (2015). A Wearable EEG-HEG-HRV Multimodal System With Simultaneous Monitoring of tES for Mental Health Management. IEEE Trans. Biomed. Circuits Syst..

[70] Simundic A.-M., Bölenius K., Cadamuro J., Church S., Cornes M.P., Van Dongen-Lases E.C., Eker P., Erdeljanovic T., Grankvist K., Guimaraes J.T. (2018). Joint EFLM-COLABIOCLI Recommendation for Venous Blood Sampling. Clin. Chem. Lab. Med. CCLM.

[71] Ojeda Del Pozo N., Del Pino Sáez R., Ibarretxe Bilbao N., Schretlen D.J., Peña Lasa J. (2016). Test de evaluación cognitiva de Montreal: Normalización y estandarización de la prueba en población española. Rev. Neurol..

[72] Craig C.L., Marshall A.L., Sjöström M., Bauman A.E., Ainsworth B.E., Pratt M., Ekelund U., Yngve A., Sallis J.F., Oja P. (2003). International Physical Activity Questionnaire: 12-Country Reliability and Validity. Med. Sci. Sports Exerc..

[73] Toba K., Okochi J., Takahashi T., Matsubayashi K., Nishinaga M., Yamada S., Takahashi R., Nishijima R., Kobayashi Y., Machida A. (2005). [Development of a portable fall risk index for elderly people living in the community]. Nihon Ronen Igakkai Zasshi Jpn. J. Geriatr..

[74] Ishimoto Y., Wada T., Kasahara Y., Kimura Y., Fukutomi E., Chen W., Hirosaki M., Nakatsuka M., Fujisawa M., Sakamoto R. (2012). Fall Risk Index Predicts Functional Decline Regardless of Fall Experiences among Community-Dwelling Elderly. Geriatr. Gerontol. Int..

[75] Yardley L., Beyer N., Hauer K., Kempen G., Piot-Ziegler C., Todd C. (2005). Development and Initial Validation of the Falls Efficacy Scale-International (FES-I). Age Ageing.

[76] Kempen G.I.J.M., Todd C.J., Van Haastregt J.C.M., Rixt Zijlstra G.A., Beyer N., Freiberger E., Hauer K.A., Piot-Ziegler C., Yardley L. (2007). Cross-Cultural Validation of the Falls Efficacy Scale International (FES-I) in Older People: Results from Germany, the Netherlands and the UK Were Satisfactory. Disabil. Rehabil..

[77] Lomas-Vega R., Hita-Contreras F., Mendoza N., Martínez-Amat A. (2012). Cross-Cultural Adaptation and Validation of the Falls Efficacy Scale International in Spanish Postmenopausal Women. Menopause.

[78] Dite W., Temple V.A. (2002). A Clinical Test of Stepping and Change of Direction to Identify Multiple Falling Older Adults. Arch. Phys. Med. Rehabil..

[79] Podsiadlo D., Richardson S. (1991). The Timed “Up & Go”: A Test of Basic Functional Mobility for Frail Elderly Persons. J. Am. Geriatr. Soc..

[80] Andersson M., Moberg L., Svantesson U., Sundbom A., Johansson H., Emtner M. (2011). Measuring Walking Speed in COPD: Test-Retest Reliability of the 30-Metre Walk Test and Comparison with the 6-Minute Walk Test. Prim. Care Respir. J..

[81] Rikli R.E., Jones C.J. (1999). Development and Validation of a Functional Fitness Test for Community-Residing Older Adults. J. Aging Phys. Act..

[82] Spagnuolo D.L., Jürgensen S.P., Iwama Â.M., Dourado V.Z. (2010). Walking for the Assessment of Balance in Healthy Subjects Older than 40 Years. Gerontology.

[83] Carlos-Vivas J., Pérez-Gómez J., Delgado-Gil S., Campos-López J.C., Granado-Sánchez M., Rojo-Ramos J., Muñoz-Bermejo L., Barrios-Fernandez S., Mendoza-Muñoz M., Prado-Solano A. (2020). Cost-Effectiveness of “Tele-Square Step Exercise” for Falls Prevention in Fibromyalgia Patients: A Study Protocol. Int. J. Environ. Res. Public Health.

[84] Jamovi The Jamovi Project 2024. https://www.jamovi.org/.

[85] Nokham R., Kitisri C. (2017). Effect of Square-Stepping Exercise on Balance in Older Adults: A Systematic Review and Meta-Analysis. J. Phys. Fit. Sports Med..

[86] Ligo Teixeira C.V., Gobbi S., Pereira J.R., Vital T.M., Soleman Hernandez S.S., Shigematsu R., Bucken Gobbi L.T. (2013). Effects of Square-Stepping Exercise on Cognitive Functions of Older People. Psychogeriatrics.

[87] Wang Y.-H., Liu Y.-H., Yang Y.-R., Wang R.-Y. (2021). Effects of Square-Stepping Exercise on Motor and Cognitive Function in Older Adults—A Systematic Review and Meta-Analysis. Geriatr. Nur..

[88] Liu-Ambrose T., Khan K.M., Eng J.J., Janssen P.A., Lord S.R., McKay H.A. (2004). Resistance and Agility Training Reduce Fall Risk in Women Aged 75 to 85 with Low Bone Mass: A 6-Month Randomized, Controlled Trial. J. Am. Geriatr. Soc..

[89] Teixeira-Salmela L.F., Santiago L., Lima R.C.M., Lana D.M., Camargos F.F.O., Cassiano J.G. (2005). Functional Performance and Quality of Life Related to Training and Detraining of Community-Dwelling Elderly. Disabil. Rehabil..

[90] Sakugawa R.L., Moura B.M., Orssatto L.B.d.R., Bezerra E.d.S., Cadore E.L., Diefenthaeler F. (2019). Effects of Resistance Training, Detraining, and Retraining on Strength and Functional Capacity in Elderly. Aging Clin. Exp. Res..

[91] Shimada H., Doi T., Lee S., Tsutsumimoto K., Bae S., Makino K., Nakakubo S., Arai H. (2022). Identification of Disability Risk in Addition to Slow Walking Speed in Older Adults. Gerontology.

[92] Beauchet O., Annweiler C., Assal F., Bridenbaugh S., Herrmann F.R., Kressig R.W., Allali G. (2010). Imagined Timed Up & Go Test: A New Tool to Assess Higher-Level Gait and Balance Disorders in Older Adults?. J. Neurol. Sci..

[93] Bakker M., de Lange F.P., Stevens J.A., Toni I., Bloem B.R. (2007). Motor Imagery of Gait: A Quantitative Approach. Exp. Brain Res..

[94] Kawabata M., Gan S.R., Shen-Hsing A.C. (2024). Effects of Square Stepping Exercise on Cognitive, Physical, Psychological, and Group Functioning in Sedentary Older Adults: A Center-Based Hybrid Trial. BMC Geriatr..

[95] Cha H., Kim K., Baek S. (2022). Square-Stepping Exercise Program Effects on Fall-Related Fitness and BDNF Levels in Older Adults in Korea: A Randomized Controlled Trial. Int. J. Environ. Res. Public Health.

[96] Hong S.-G., Kim J.-H., Jun T.-W. (2018). Effects of 12-Week Resistance Exercise on Electroencephalogram Patterns and Cognitive Function in the Elderly With Mild Cognitive Impairment: A Randomized Controlled Trial. Clin. J. Sport Med. Off. J. Can. Acad. Sport Med..

[97] Lin S.-Y., Jao C.-W., Wang P.-S., Liou M., Wu J.-L., Chun H., Tseng C.-T., Wu Y.-T. (2021). Differences in Physiological Signals Due to Age and Exercise Habits of Subjects during Cycling Exercise. Sensors.

[98] Jeong J.-S., Yu M., Kwon T.-K. (2021). Effect of Lower Limb Exercise on Posture Stability and Brain Activity during Whole Body Vibration for the Elderly. J. Mech. Med. Biol..

[99] Cabral D.F., Rice J., Morris T.P., Rundek T., Pascual-Leone A., Gomes-Osman J. (2019). Exercise for Brain Health: An Investigation into the Underlying Mechanisms Guided by Dose. Neurother. J. Am. Soc. Exp. Neurother..

[100] Park H., Ishigami A., Shima T., Mizuno M., Maruyama N., Yamaguchi K., Mitsuyoshi H., Minami M., Yasui K., Itoh Y. (2010). Hepatic Senescence Marker Protein-30 Is Involved in the Progression of Nonalcoholic Fatty Liver Disease. J. Gastroenterol..

[101] Yamaguchi M., Neale Weitzmann M., Murata T. (2012). Exogenous Regucalcin Stimulates Osteoclastogenesis and Suppresses Osteoblastogenesis through NF-κB Activation. Mol. Cell. Biochem..

